# Different Perceptions among Women and Their Physicians Regarding Contraceptive Counseling: Results from the TANCO Survey in Brazil

**DOI:** 10.1055/s-0040-1712145

**Published:** 2020-05

**Authors:** Rogério Bonassi Machado, Thaís Emy Ushikusa, Ilza Maria Urbano Monteiro, Cristina Aparecida Falbo Guazzelli, Zsuzsanna Jarmy di Bella, Carlos Alberto Politano, Luís Carlos Sakamoto

**Affiliations:** 1 Department of Obstetrics and Gynecology, Faculdade de Medicina de Jundiaí, Jundiaí, SP, Brazil; 2 Bayer, São Paulo, SP, Brazil; 3 Department of Obstetrics and Gynecology, Universidade Estadual de Campinas, Campinas, SP, Brazil; 4 Department of Obstetrics, Escola Paulista de Medicina, Universidade Federal de São Paulo, São Paulo, SP, Brazil; 5 Department of Gynecology and Obstetrics, Women's Health Reference Center, Hospital Pérola Byington, São Paulo, SP, Brazil

**Keywords:** contraception, counseling, knowledge, decision making, contracepção, aconselhamento, conhecimento, tomada de decisão

## Abstract

**Objective** The optimal use of contraceptive methods requires that women participate in targeted choice of methods that meet their individual needs and expectations. The Thinking About Needs in Contraception (TANCO) study is a quantitative online survey of the views of health professionals and women on aspects of contraceptive counseling and contraceptive use.

**Methods** Physicians and women attending clinics for contraception were invited to complete online questionnaires. The research explored the knowledge and use of contraceptive methods, satisfaction with the current method and interest in receiving more information on all methods. Aspects related to contraceptive practice among physicians were gathered in parallel. The results obtained in the Brazilian research were compared with those of the European research, which involved 11 countries.

**Results** There was a high prevalence of contraceptive use and general satisfaction with the current method. A total of 63% of the women were using short-acting contraceptive (SAC) methods, and 9% were using a long-acting reversible contraceptive (LARC). Sixty-six percent of women were interested in receiving more information on all methods; 69% of women said they would consider LARC if they received more comprehensive information about it. Health professionals tend to underestimate the interest of women in receiving information about contraception in general, and more specifically about LARCs.

**Conclusion** Despite the high levels of use and satisfaction with the current methods, women were interested in receiving more information on all contraceptive methods. Structured contraceptive counseling based on individual needs and expectations may lead to greater knowledge and a greater likelihood of proper contraceptive choice.

## Resumo

**Objetivo** A utilização ideal de métodos contraceptivos requer que as mulheres participem da escolha orientada sobre métodos que atendam às suas necessidades e expectativas individuais. O estudo *Thinking About Needs in Contraception* (TANCO)—Pensando nas Necessidades em Contracepção—é uma pesquisa quantitativa online de opiniões de profissionais de saúde e de mulheres sobre aspectos do aconselhamento contraceptivo e uso de anticoncepcionais.

**Métodos** Médicos e mulheres que frequentam consultórios de atendimento visando contracepção foram convidados a preencher questionários online. A pesquisa explorou o conhecimento e o uso de métodos contraceptivos, a satisfação com o método atual, e o interesse em receber mais informações sobre todos os métodos. Aspectos relacionados à prática contraceptiva entre médicos foram reunidos em paralelo. Os resultados obtidos na pesquisa brasileira foram comparados aos da pesquisa europeia, que envolveu 11 países.

**Resultados** Houve alta prevalência do uso de contraceptivos e satisfação geral com o método atual. Sessenta e três por cento das mulheres estavam usando métodos de curta duração (SAC, na sigla em inglês), e 9% estavam usando um método de longa ação (LARC, na sigla em inglês). Sessenta e seis por cento das mulheres estavam interessadas em receber mais informações sobre todos os métodos; 69% das mulheres disseram que considerariam um LARC se recebessem informações mais abrangentes sobre esse método. Os profissionais de saúde tendem a subestimar o interesse das mulheres em receber informações sobre a contracepção em geral e, mais especificamente, sobre os LARC.

**Conclusão** Apesar dos altos níveis de uso e satisfação com os métodos atuais, as mulheres estavam interessadas em receber mais informações sobre todos os métodos contraceptivos. O aconselhamento contraceptivo estruturado, baseado nas necessidades e expectativas individuais pode levar ao maior conhecimento e à maior probabilidade de escolha contraceptiva adequada.

## Introduction

In face of the wide variety of contraceptive methods available, the accurate guidance with emphasis on the individual needs of women, particularly related to the lifestyle, risk factors, additional benefits, and efficacy, becomes important.^1^ ^2^ Modern contraceptive counseling has as its main pillars the focus on the woman as a core element and the encouragement of the individual discussion, allowing the method to be chosen by means of shared decision.^3^ ^4^ However, despite of the growing number of contraceptive method users and available variety, several women are still subject to unplanned pregnancies and their consequences. Estimates in the United States indicated that half of the female population who suffer an unplanned pregnancy mention the use of a contraceptive method in the month it occurred.^5^ Trussell et al^6^ noted that, in that country, the unplanned pregnancy rates were 9% during the 1^st^ year of typical use for all combined hormonal contraceptives (CHCs).^6^ In Brazil, contraceptive use prevalence is 76.7%, with 62.7% of these aiming to limit the number of children and 14% to space births.^7^ However, in the poorest regions of the country, the reported prevalence of contraceptive use reaches only 62%, as a result of lower access to family planning services.^8^ In addition to difficult access to contraceptive methods, the uncompliant use is particularly important with respect to the failure rate of each method. In general, low compliance relates to poor awareness of the method characteristics, and can be associated to the lack of motivation for use and, further, to the woman dissatisfaction with the chosen method.^9^ ^10^

The North-American CHOICE study showed that the increased knowledge and the removal of financial barriers led to an increased use, satisfaction, and continuation rate of long-acting reversible contraceptives (LARCs).^11^ ^12^ As a result, international entities incorporated the recommendation of LARCs as the first line of choice among all contraceptive methods.^12^ ^13^ ^14^ ^15^ ^16^ On the other hand, healthcare professionals in Brazil, particularly gynecologists, are still the more reliable source of information concerning sexual health, and, therefore, they are in a core position for contraceptive counseling, despite of the global increase in the use of internet and social media.^17^

A German study directly investigated to what extent the choice of the contraceptive method by women depends on their gynecologists and if more extensive counseling could increase the interest in using a LARC.^18^ Only 9% of the 18,521 women used LARCs; however, 60% of the women would consider LARCs as an option if they received more information about it. The gynecologists, on the other hand, underestimated such interest, believing that only 18% of the women would be interested in LARCs.^18^

The Thinking About Needs in Contraception (TANCO) study was a multinational online survey intended to identify the relationship between women and contraceptive methods, in addition to the opinions and insights of physicians concerning the subject. Brazil and 11 European countries joined the study. By assessing women and physicians in parallel, the main objectives of the TANCO study were contraceptive use and awareness, satisfaction with the current method, in addition to identifying the needs and expectations of women with respect to contraception as well as their interest in receiving more information about all methods.

This publication describes the results of the Brazilian TANCO study and makes comparative considerations with the European results.

The main objectives of the TANCO study in Brazil were:

To evaluate women's awareness and self-reported knowledge of contraception;
To detect which contraceptive is most frequently recommended by physicians and preferred by women;
To assess satisfaction and compliance with the current method;

To evaluate how physicians are estimating their own performance in terms of contraceptive counseling and offered services compared with the women's perception.

## Methods

In Brazil, the TANCO study was a quantitative online survey about the gynecologists' and their patients' points of view concerning counseling aspects with respect to contraception and their use. The study concept was designed along with a global market survey organization (Psyma Health and Care) and the sponsor, Bayer SA.

Gynecologists with at least 2 years of clinical experience and providing regular contraceptive counseling services were selected from a market survey database. Women between 18 and 49 years old were invited to join the survey by their own physicians. The written information containing the study description and instructions on how to access the online questionnaire were delivered to the selected women, who had to agree to participate.

The study involved the completion of anonymous online questionnaires, the starting point of which was a 45-item survey developed by contraception specialists in Germany for use in the German TANCO study described by Oppelt et al^18^ The Brazilian TANCO study questions were extracted, modified, and evaluated for scientific quality, clarity, and relevance to the Brazilian population. An overview of the topics included in the Brazilian TANCO study is in Table 1. The final questionnaires included 18 questions for women and 17 questions for healthcare professionals and were elaborated to take 10 to 15 minutes to be completed, respectively.

**Table 1:** Topics included in the Brazilian TANCO study questionnaire

Topics	Content	Physician	Woman
	Workplace	√	x
	Experience	√	x
General scenario	Number of treated patients	√	x
	Marital status/parity	x	√
	Expectations concerning contraception	x	√
	Frequently recommended/requested contraceptive methods	√	x
	Experience with IUD	√	x
Contraception:	Knowledge about the available methods	x	√
- Knowledge	Current use/preference of contraceptives	x	√
- Use	Pill users: personal experience	√	√
- Satisfaction	Pill forgetting frequency	√	√
	Experience with emergency contraception	√	√
	Satisfaction level with the contraceptive method	√	√
	Level of interest to receive more information about LARCs	√	√
	Frequency of visits to discuss family planning	√	√
Evaluation of the contraception consultation	General evaluation of the contraceptive counseling	√	√
	Wish to provide/receive more frequent or more extensive guidance	√	√
	Satisfaction with punctuality	√	√
	Satisfaction with the waiting time	√	√
Service evaluation	Attendance team friendliness	√	√
	Professionalism during the consultation	√	√
	Time spent with clarification	√	√
	Other services (reminders, etc.)	√	√

Abbreviations: IUD, intrauterine device; LARC, long-acting reversible contraceptive; TANCO, Thinking About Needs in Contraception.

The self-reported knowledge regarding contraceptive methods were evaluated using a four-point scale: “I know it very well”; “I have basic information”; “I've just heard about it”; “I've never heard about it.” The satisfaction with the current method and contraceptive needs were evaluated using a 7-point scale (1 = not satisfied at all/not relevant and 7 = very satisfied/very relevant). The interest in receiving more detailed or frequent information was based on the four-point scale: “I'm interested in knowing more, particularly about new products”; “Yes, more extensive”; “Yes, more frequent and extensive”; “No, I don't need further information.” The last part of the evaluation verified the patient's point of view concerning contraceptive counseling services provided by her own physician, including cordiality, time spent, and quality, among others, using a 7-point scale being 1 “insufficient/poor” to 7 “very good.”

The study took part from February to November 2017. No approval of the research protocol or of the informed consent form was needed since the data was anonymized, aggregated and only numerical in the reports, and not retained by the market research agency. Psyma, using SPSS, conducted the statistical analysis of the data. Participating physicians received a monetary incentive proportional to their dedicated time, and non-monetary (their patients' answer report, numerical only) according to local market research regulations.

## Results and Discussion

### Characteristics of the Study Population

From February to November 2017, 50 Brazilian physicians and 1,113 women answered the online questionnaire. Among the physicians, 78% were gynecologists and obstetricians, and 22% were only gynecologists. Sixty-six percent of the physicians were female. All of them worked in a private practice, and 12% mentioned they also worked in a hospital setting. These physicians had 26 years of experience on average in the profession. A total of 1,113 women were enrolled in the study, with an average age of 32 years old. Forty-two percent of the sample corresponds to the age group from 18 to 29 years; 39% from 30 to 39 years; and 19% corresponds to between 40 and 49 years of age. Fifty-six percent of them were married or on a stable relationship; 74% of them were employed on a full-time or part-time job. Almost half of the women were mothers, with an average of 2 children. Concerning family planning, at the time of the interview, only 4% of the women were currently pregnant or planning to have children, and 3% mentioned they were planning to get pregnant in the next 2 years. The majority of the women, 81%, was not planning to have children within 5 years.

### Contraceptive Methods Knowledge

**Fig. 1:**
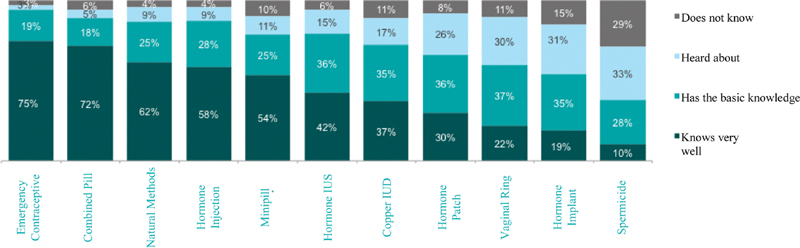
Level of knowledge of Brazilian women concerning different contraceptive methods. Abbreviations: IUS, intrauterine system; IUD, intrauterine device.

The physicians believed that half of their patients had a poor knowledge about the vaginal ring and the subcutaneous implant, and about ⅓ of them believed that their patients had a good knowledge about the intrauterine hormonal and non-hormonal devices. Concerning their customary monthly prescription, 63% correspond to pills, 11% to levonorgestrel-releasing intrauterine device, and 6% to copper intrauterine device. In their turn, around 70% of the interviewed women reported having good information about the pills, while only 40% have some knowledge about long-acting methods, such as the intrauterine hormonal device (Fig. 1). It is worth noting that regardless of the different age groups, whether married or not, nulliparous or not, the knowledge concerning the pills is around 70%. On the other hand, the level of knowledge concerning the intrauterine hormonal device is higher in women above 30 years of age and married compared with the single ones in the age group from 18 to 29 years old.

Noticeably, women have good knowledge concerning the short-acting methods such as daily pills and also about the morning-after pill; however, the long-acting reversible methods are less known and considerably less used.^19^

It is worth mentioning that the emergency contraception pill is the best-known contraceptive method (75%), which, in our country, consists of using 1.5 mg of levonorgestrel through oral route as a single dose or 2 fractioned doses with a 12 hour interval. Although there is a regulation concerning the need of a medical prescription, it is freely marketed in drugstores, which makes it extremely accessible for women.^7^

Undoubtedly, the use of emergency contraception significantly reduces the number of unplanned pregnancies, but it should not be the most commonly used method because its efficacy is significantly lower than the other short or long-acting contraceptive methods.

Another interesting aspect is that, despite the good self-reported knowledge about intrauterine hormonal (42%) and non-hormonal (37%) devices by women, these methods correspond only to 11% and 6% of the medical prescriptions in this study, respectively.

The fact that more than half of the interviewed women do not intend to have children in a period of at least 5 years is noteworthy. In spite of that, only 9% of them use a LARC, such as the levonorgestrel-releasing intrauterine system (6%), copper IUD (2%), or subcutaneous implant (1%). This aspect clearly shows the need for an improved medical training in contraceptive counselling and LARC's insertions.^14^

### Use and Satisfaction with the Contraceptive Method

**Fig. 2:**
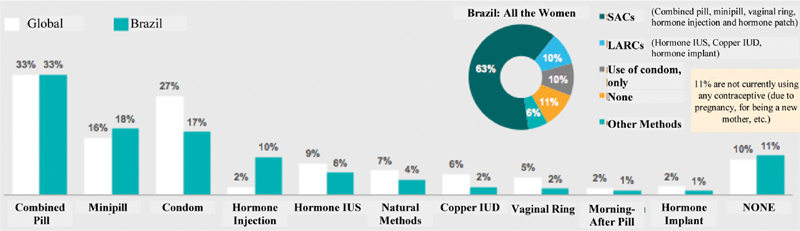
Contraceptive methods used by women in Brazil and 11 European countries (Global). Abbreviations: LARC - long-acting reversible contraceptive; SAC, short-acting contraceptive; IUS, intrauterine system; IUD, intrauterine device.

Fig. 2 shows the frequency of contraceptive methods used by Brazilian and European women. Sixty-three percent of women use a short-acting method, predominantly combined oral contraceptive (COC) (33%). Long-acting reversible contraceptives are still underused, only 10% in Brazil, which is the same percentage of women who do not use any contraceptive method.

**Fig. 3:**
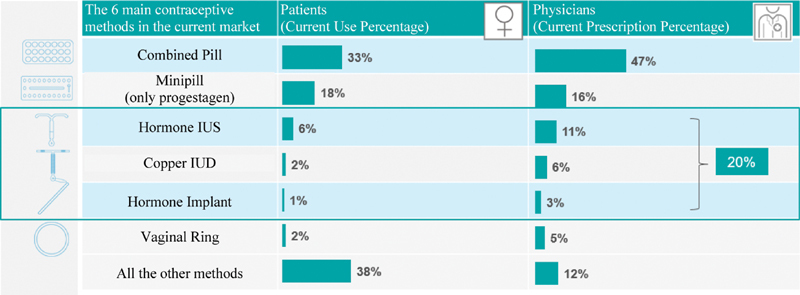
Comparison between the utilization rate of contraceptive methods and prescription rate.

Regarding medical prescriptions, in Brazil COCs are the most commonly prescribed method, on average 47% of the times, followed by the progestogen only pill (POP) (16%), IUS (11%), IUD (6%), vaginal ring (5%), injectable (5%), implant (3%), and patch (3%) (Fig. 3). On average, physicians serve 184 women between 18 and 49 years old for contraceptive counseling per month, and, generally, 30 IUSs and 25 IUDs are implanted per year.

**Fig. 4:**
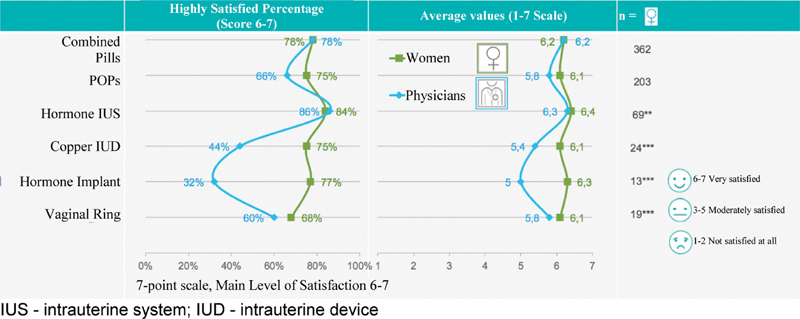
Level of satisfaction with the method currently used by women in Brazil. Abbreviations: IUS, intrauterine system; IUD, intrauterine device.

The satisfaction level with the current contraceptive method is shown in Fig. 4. A great difference of perception is noted concerning the satisfaction with the implant, copper IUD, POP, and vaginal ring. Women are generally more satisfied with their current methods than their physicians expected. The satisfaction with the IUS is the highest among all methods, as expected by physicians.

Although medical prescriptions for COCs are ∼ 50% of the total, by using the satisfaction scale graduated from 1 to 7, we noted that more than 70% of the women are very satisfied with all hormonal methods. When we compared the satisfaction among the different types of LARCs, we noted that it was higher with the hormonal IUS (84%) than with the copper IUD (75%) and implant (77%).

In the physicians' perception, the satisfaction degree 6 and 7 (very satisfied) is approximately doubled with hormonal IUS = 86% compared with copper IUD = 44%, and more than double compared with the implant (32%).

When asked about the main desired attributes of any contraceptive method, women answered efficacy and safety (good reliability and lower risk of thrombosis in 93% and 94%, respectively), characteristics related to the LARCs. However, when we analyzed their use in Brazil compared with other participating countries, we noted that less than half (34% against 66% of the global total) of women used LARCs. Our low utilization rate leads us to think that implementing governmental and institutional measures to raise reproductive and family planning awareness will ultimately lead to an increased use of LARCs and, consequently, a decrease in the unplanned pregnancy rate in our country.

Despite the small sample, when we analyzed the use of LARCs compared with the age, we realized that 46% of the users are in the age group of 30 to 39 years, and the extremes are equally distributed. As for the marital status, there is a higher frequency of LARCs users among married women, with 60% using IUS and 69% using implant, while only 41% chose the IUD.

### Uncompliant Use of the Short-acting Methods

**Fig. 5:**
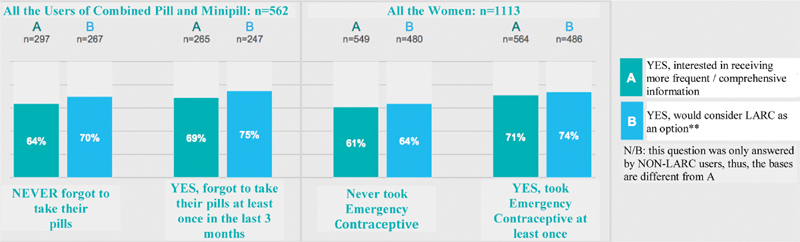
Inappropriate use of oral contraceptives among Brazilian women.

Specifically analyzing COCs, 47% of the users mentioned they forgot to take one or more pills in the 3 months prior to the study (Fig. 5). Combined oral contraceptives are effective, but they require regular pill intake. A daily routine can be difficult for a great number of women and favor a discontinuity of the method.

The data from the Brazilian TANCO study are similar to those found in the literature, confirming that the uncompliant use leads to a theoretical decrease in efficacy, as already shown in some studies, in which the failure rate described as “typical/routine use” reaches 9% a year.^6^ ^20^ Any forgotten pill may, however, contribute to an increased risk of unplanned pregnancy and cause uncertainty among their users. For women who reported to have forgotten 3 or more pills (17%) in the 3 months prior to the study, the risk is even higher.^21^

The studies mention that forgetting 1 to 3 pills per cycle is a frequent problem that occurs with 15 to 51% of the users, particularly among adolescents, probably due to the young age, inability to establish a routine, unavailability of the method, side effects, loss of motivation, and lack of involvement in the final decision of COCs.^22^

Among interviewed Brazilian women, particularly those younger than 30 years of age, missing the pill was the reason for nervousness (38%) and great concern with the consequences as a result from this act (25%). The use of methods that do not rely on the user could prevent those feelings.

Most women, who chose COCs as contraceptive method, despite referring a high level of satisfaction, mention that the daily intake is a concern, resulting in the irregular and eventually inefficient use.

Maybe this explains the fact that most of the oral hormonal contraceptive users, regardless of the intake routine, wanted to receive more information about other contraceptives such as LARCs, for example. Another relevant result is that ∼ 75% of women who forgot to take the pill, after receiving guidance and information from their physicians would consider LARCs as an option.

Our results show great interest in knowing more about LARCs, as reported by the CHOICE project, in which 75% of the subjects chose 1 of the 3 methods following counseling.^23^ Previous researches showed that most women have a poor knowledge concerning other contraceptive options, particularly the most effective ones.^24^ ^25^

A study about the Contraceptive Use, Pregnancy Intention and Decisions (CUPID) conducted with 3,795 Australian women between 18 and 23 years of age noted the need for consistent and accurate information concerning all contraceptive methods in addition to those usually provided about the oral hormonal methods. The authors described that, many times, the patients felt frustrated due to the limitations posed by their physicians or healthcare professionals, particularly when they are younger.^26^

In general, healthcare professionals believe that there is a lack of interest by their patients concerning information about all contraceptive methods, and this can be a limiting factor for the use of LARCs.^15^ ^27^

In our study, 56% of the gynecologists believed that the women used the oral method inconsistently, forgetting 1 or more pills in the previous three months of use. Having this in mind, why not offer other methods?

Studies have showed that physicians believe in “presumed knowledge,” that is, that the women already have information and data concerning such methods, and, thus, there is no need for a comprehensive presentation or discussion about it.^25^ ^28^ ^29^ The authors conclude that such factors are limiting for LARCs use, and that physicians and healthcare professionals have an important role in increasing the awareness of women, particularly the younger ones, about the most effective methods.^28^ ^29^

### Emergency Contraception

In our study, 51% of the women mentioned they have already used at least once the emergency contraception (EC), regardless of the age group or marital status. Thirty percent mentioned to have used this method two or three times, and it was associated to nervousness and great concern every time. In contrast, physicians estimated that only 38% of the patients had used the EC at least once, indicating that pill compliance is worse than expected by their physicians or an increased uncertainty with the method by the users. Young and single women (25% and 35%, respectively) rely on EC more frequently than those older and married (12% and 8%, respectively).

Emergency contraception provides a mean to decrease unplanned pregnancy after having unprotected sexual intercourse and must not be considered as a replacement for regular effective contraception. The easy access to this method leads to an increased use, but it does not result in a significant decrease in pregnancy rates of the general population.^30^ ^31^ ^32^ The authors of these studies confirm the importance of knowledge and the use of highly efficient methods.

Different perceptions between physicians and patients also occur when the subject is what to do after missing one pill. More than 90% of the physicians believe that 41% of the patients seek for counseling regarding what to do when in this case and state that 30% of them change their contraceptive method after using EC. This thought is not in line with what the women really do; only 8% of women sought their physicians after forgetting the pill, 6% sought for information on the internet, and only 1% consulted with the pharmacist. As for attitude, 46% of them take the missed pill along with the next one, without adopting any other precaution to avoid pregnancy. Twenty-three percent of the women did not change their attitude, 19% used EC, and 28% used condoms, abstinence, or *coitus interruptus* until the next menstrual period.

### Expectations of the Consultation Concerning Contraception

**Fig. 6:**
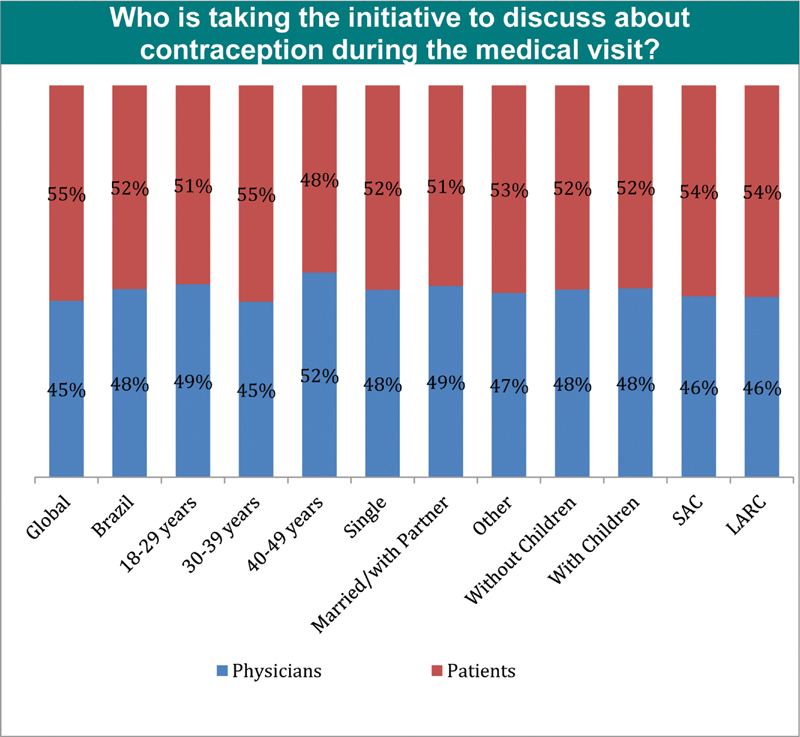
Perception according to the women concerning the initiative about contraception. Abbreviations: LARC, long-acting reversible contraceptive; SAC, short-acting contraceptive.

**Fig. 7:**
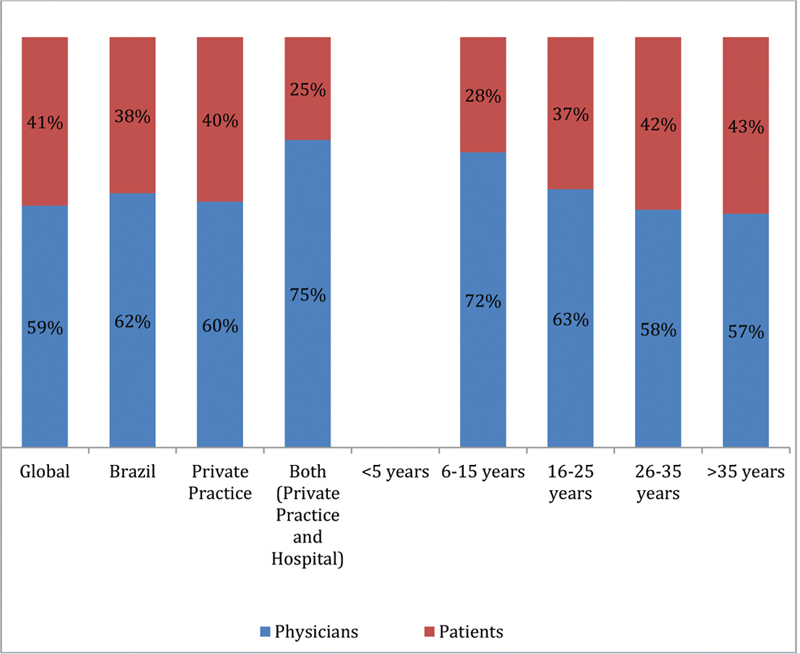
Perception according to the physicians concerning the initiative about contraception. Abbreviation: Years - Years of experience.

The results showed that according to the women, more than 50% of them are responsible for initiating a discussion concerning contraception both in the global study (55%), and in Brazil (52%) (Fig. 6). However, when physicians were asked who initiates the contraceptive counselling; the results showed that 62% starts with the physicians, similarly to the results of the global study (Fig. 7).

In the Brazilian TANCO study, the physicians' profile was similar to those of the European study.^33^ The different perceptions between patients and physicians regarding who is taking the initiative to discuss contraception is reflected in other situations, in which the healthcare professionals tend to underestimate the women's interest in receiving information concerning contraception in general and, more specifically, about LARCs.^21^ Also, the low self-reported awareness concerning the methods suggest that women may rapidly discard LARCs as inappropriate during the counseling process or be unable to choose the most appropriate method due to access restrictions.^33^

In general, the women are satisfied or very satisfied with their physicians and with the services provided by them, the level of satisfaction is higher for friendly staff and quality of the consultation.

### Interest in Information Concerning Contraceptive Methods

Despite of the high satisfaction rates with the current method, 61% of the women wish to know more about other contraceptive methods, often times more than one method, on a more regular and comprehensive basis, especially women under 40 years of age (70%) and without children (69%). Many women also showed interested in receiving information about methods with low hormonal dose (76%). The high satisfaction rates found in the study are not surprising, once the unsatisfied women probably changed the method previously.^33^

The first 3 methods of interest were the subdermal implant, the transdermal patch, and the intrauterine hormonal system, with 22%, 15%, and 15%, respectively. Such interest is higher among women who forgot to take the pill in the previous 3 months or who have already used EC. In addition, more than 90% of the women are interested in using contraceptive methods with low hormonal dose regardless of the age, marital status, parity, or current method.

Physicians believe that the patients' knowledge about contraceptive methods is high regarding IUD (94%) and IUS (100%), but it falls by half in regards to the implant (52%) and vaginal ring (48%). Women are more satisfied using COCs (78%) and the IUS (86%), compared with the IUD (44%) and the implant (32%). Physicians assume that 55% of the women wish to receive information concerning several contraceptive methods, with 49% being interested in information about the short-acting methods and 55% in the LARCs, making them an interesting choice.

For women the 3 key attributes of a contraceptive method are 1) having low or no risk of thrombosis, 2) high efficacy 3) protection against sexually transmitted infections with 94%, 93%, and 89%, respectively. Such values decrease as the age advances (95% between 18 and 29 years versus 87% between 40 and 49 years) but does not differ with lifestyle such as marital status, parity, or use of current method such as pill or LARC, because they depend on the individuals needs of each of them. For the physicians, the three mandatory attributes of contraceptive are having low risk of thrombosis, high efficacy and reduction of the menstrual bleeding and pain with 100%, 98% and 98%, respectively.

Diving deeper into the women's needs and expectations regarding contraception could lead to an increased knowledge, more efficient discussions with healthcare professionals, and better-informed contraceptive choice. As a result, by receiving more extensive information, 73% of women could use LARCs.^21^

### Interest in Information Concerning LARCs

The small number of women who use LARCs compared with women who use oral contraceptives is surprising, since most women desire a very reliable contraceptive method, preferably one that does not rely on the user's compliance. For this reason, 60% of them stated that LARCs would be an option if they had more information about it, compared with 18% of the gynecologists who believed in such statement, underestimating the patients' interest.

The importance of counseling was exposed in the CHOICE project, in which the vaginal ring utilization rates increased from 7.5 to 21.8%, and the patch from 3.3 to 5.8%. The more extensive knowledge of contraceptive methods in general allowed one third of women to choose a different contraceptive method than they initially thought of.^34^ ^35^ It is well known that family situation, age, and profession motivate the choice of the contraceptive method.^36^ ^37^

The demand for more knowledge about contraceptive methods was evident in the fact that, in the study, ¾ of the women considered switching to LARCs if they received proper information from the healthcare professionals regarding efficacy and safety. Sixty-seven percent of them reported an interest in receiving more extensive and frequent information about all contraceptive methods, and this number goes up to 84% among women who were not planning to have children in the next 5 years.

**Fig. 8:**
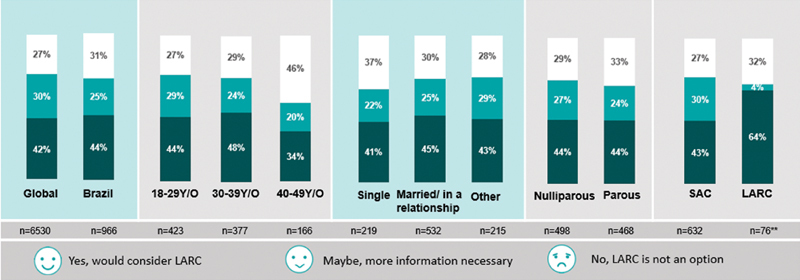
Interest in considering long-acting reversible contraceptive as an option if the women received more information from their physicians.

Concerning parity, half of the women who have never been pregnant mentioned that they could consider using a LARC, but they would require more information. When dividing women into age groups, half of the women between 18 to 39 years would consider LARCs as an option. One quarter of these Brazilian women mentioned they wished more information about the contraceptive methods (Fig. 8).

The questionnaires used in the Brazilian TANCO study were virtual; thus, professionals needed to access and answer them online, limiting the group by willingness to join the study. As for the women, only those who sought their physician were included in the study. The lack of information can be even higher among women who do not seek health services.

Another relevant point is that the contraceptive self-knowledge was evaluated by an online test completed by the woman herself, being impossible to confirm if such referred knowledge is translated into actual knowledge.

Age, gender, type of healthcare professional, and experience with the previously used contraceptive method influence in the extension of the discussion of some methods (IUD or pill, for example).^36^ In our study, only women older than 18 years of age were included, and adolescents younger than such age represent nowadays one of the groups more concerned due to the great impact of an unplanned pregnancy in this population. Another limitation is that only gynecologists joined the study, and many times nurses or other healthcare professionals are the ones responsible for counseling and offering contraceptive methods. The results about the professionals could be different if the proportion of other healthcare professionals were different.

## Conclusions

The Brazilian TANCO study showed that there is a significant coverage of the contraceptive methods among the women; however, in our study, most of them still use methods considered as being less efficient and EC. Only 9% of women use any type of LARC. Despite the low Utilization rate found in our study, it is even higher than the one seen in our country. Therefore, there is a great need to increase the knowledge regarding highly-effective methods, such as LARCs. In spite of the high satisfaction rates with the current method, women were interested in receiving further information about all contraceptive methods, particularly about LARCs. This represents a great opportunity for the healthcare professional to discuss their use, especially because women often have limited knowledge about such methods. When choosing a contraceptive, women considered safety and efficacy as the two most important attributes. More attention to individual needs and expectations may lead to an increased knowledge, more efficient discussions, and increased probability to choose the right contraceptive for each individual woman, leading to higher satisfaction rates and continuation and, ultimately, contributing for the reduction of the unplanned pregnancy rates in our country.
